# T2-Signal of Ulnar Nerve Branches at the Wrist in Guyon’s Canal Syndrome

**DOI:** 10.1371/journal.pone.0047295

**Published:** 2012-10-10

**Authors:** Jennifer Kollmer, Philipp Bäumer, David Milford, Thomas Dombert, Frank Staub, Martin Bendszus, Mirko Pham

**Affiliations:** 1 Department of Neuroradiology, University of Heidelberg, Heidelberg, Germany; 2 Division of Experimental Radiology, Department of Neuroradiology, University of Heidelberg, Heidelberg, Germany; 3 Center for Peripheral Neurosurgery, Dossenheim/Heidelberg, Germany; School of Biomedical Sciences, The University of Queensland, Australia

## Abstract

**Objective:**

To evaluate T2-signal of high-resolution MRI in distal ulnar nerve branches at the wrist as diagnostic sign of guyon’s-canal-syndrome (GCS).

**Materials and Methods:**

11 GCS patients confirmed by clinical/electrophysiological findings, and 20 wrists from 11 asymptomatic volunteers were prospectively included to undergo the following protocol: axial T2-weighted-fat-suppressed and T1-weighted-turbo-spin-echo-sequences (3T-MR-scanner, Magnetom/Verio/Siemens). Patients were examined in prone position with the arm extended and wrist placed in an 8-channel surface-array-coil. Nerve T2-signal was evaluated as contrast-to-noise-ratios (CNR) from proximal-to-distal in ulnar nerve trunk, its superficial/sensory and deep/motor branch. Distal motor-nerve-conduction (distal-motor-latency (dml)) to first dorsal-interosseus (IOD I) and abductor digiti minimi muscles was correlated with T2-signal. Approval by the institutional review-board and written informed consent was given by all participants.

**Results:**

In GCS, mean nerve T2-signal was strongly increased within the deep/motor branch (11.7±4.8 vs.controls:−5.3±2.4;p = 0.001) but clearly less and not significantly increased in ulnar nerve trunk (6.8±6.4vs.−7.4±2.5;p = 0.07) and superficial/sensory branch (−2.1±4.9vs.−9.7±2.9;p = 0.08). Median nerve T2-signal did not differ between patients and controls (−9.8±2.5vs.−6.7±4.2;p = 0.45). T2-signal of deep/motor branch correlated strongly with motor-conduction-velocity to IOD I in non-linear fashion (R^2^ = −0.8;p<0.001). ROC-analysis revealed increased nerve T2-signal of the deep/motor branch to be a sign of excellent diagnostic performance (area-under-the-curve 0.94, 95% CI: 0.85–1.00; specificity 90%, sensitivity 89.5%).

**Conclusions:**

Nerve T2-signal increase of distal ulnar nerve branches and in particular of the deep/motor branch is highly accurate for the diagnostic determination of GCS. Furthermore, for the first time it was found in nerve entrapment injury that T2-signal strongly correlates with electrical-conduction-velocity.

## Introduction

Guyon’s canal syndrome (GCS), the entrapment ulnar neuropathy at the wrist, is rare but still the second most frequent entrapment syndrome of the ulnar nerve [1]. It is either caused by identifiable compressive mass lesions (e.g. ganglion cysts) or occurs as non-tumorous entrapment neuropathy in Guyon’s canal, often due to repetitive mechanical stress to the distal ulnar nerve like in handlebar palsy in cyclists [2], [3]. Compression injury mainly affects the deep motor branch along its course either under the fibrotic arch of the flexor and abductor digiti minimi muscle, or under the pisohamate ligament [4]–[7]. To determine the presence and spatially localize nerve lesions are arguably the most important pieces of diagnostic information in patients with suspected peripheral neuropathies [1]. GCS patients typically present with motor and sometimes sensory deficits in the distribution of distal ulnar nerve branches. However, clinical and electrophysiological findings may remain ambiguous with regard to the exact site of the underlying lesion. In suspected focal neuropathies a causative mass lesion (e.g. nerve sheath tumors, ganglion cysts) as indirect diagnostic sign should be ruled out by MRI. However, direct diagnostic signs of neuropathy visualizing nerve lesions in the absence of indirect signs such as nerve T2-signal are still poorly understood and their potential diagnostic value for most peripheral neuropathies has yet to be determined [8]–[15]. Employment of MRI with particular emphasis on increased structural resolution and optimized nerve T2 contrast as applied in this study, may be termed MR Neurography (MRN) [16]–[21]. Nerve T2-signal is known to increase after various mechanical and non-mechanical forms of experimental nerve injury [16], [20] and equally has been reported to occur in a variety of focal, e.g. in carpal and cubital tunnel syndrome [4], [9], [13]–[14], but also in non-focal clinical neuropathy syndromes [22], [23], [24].

The aim of our study was to evaluate T2-signal in distal ulnar nerve branches at the wrist, which are at the limit of spatial resolution, as novel diagnostic sign of GCS and precisely map the proximal-to-distal course of nerve T2-signal in patients and controls. In addition, we correlated motor-nerve-conduction with T2-signal, an analysis which has not been performed before in any human or experimental study and which is essential to understand the electrophysiological/functional relevance of nerve T2-signal increase.

## Materials and Methods

### I. Study Design and Ethics Statement

All participants gave written informed consent and the study was approved by the institutional ethics board (university of Heidelberg; S-057/2009). Eleven patients (4 male: mean age 57.3, range 43–68; 7 female: mean age 47.3, range 24–74; total: mean age 50.9, range 24–74) with confirmed GCS and 20 control wrists from 11 asymptomatic volunteers (3 male: mean age 33.3, range 33–34; 8 female: mean age 28.5, range 21–51; total: mean age 29.8 years, range 21–51) were prospectively included in the study. Physical, electrophysiological and MRI examinations took place between January 2010–May 2011. In patients with GCS the mean time gap between MRI and electrophysiological measurements was 14d±8. Due to limited examination slots for a detailed clinical/electrophysiological assessment, the time between MRI and clinical/electrophysiological evaluation was longer in the group of healthy volunteers (mean of 60d±15).Volunteers were included if they had no history of acute/chronic upper extremity or cervical pain or surgery, and no paresthesia or motor deficits in the upper extremities. Further exclusion criteria were any chronic metabolic, malignant or inflammatory illness, and any history of neuropathic symptoms.

### II. Ulnar Nerve Anatomy at the Wrist

The anatomy of Guyon’s canal and branching patterns of the ulnar nerve including variations of its bifurcation into deep motor and superficial sensory branch are well known [25]–[34]. According to Shea and McClain [35] and Gross and Gelbermann [36], guyon’s canal may be subdivided into three zones: I) Proximal to pisiform bone (bifurcation site), II) deep/motor branch compartment, III) superficial/sensory branch compartment.

For precise image data analysis nerve T2-signal was mapped from proximal-to-distal with anatomical registration to the center of the pisiform bone as the center of guyon’s canal that was assigned a reference position of “0? (distance to center  = 0 mm). Slices proximal to this landmark were labeled with decreasing negative numbers, slices distal to center with increasing positive numbers. Ulnar nerve trunk was evaluated from slice positions -9 through 0 mm; T2-signal of motor and sensory branches was evaluated on positions +1 to +23 mm. As internal control, T2-signal of median nerve was determined at positions −9 to +23 mm. The first and last slices were excluded from analysis because of artificial signal loss related to coil sensitivity profile in z-direction.

### III. Clinical and Electrophysiological Examination

Clinical and electrophysiological evaluation in GCS patients and in healthy volunteers were always performed by T.D. and F.D. (board certified neurosurgeon and neurologist each with more than 20 years of experience in diagnosing and treating peripheral neuropathies). Electrophysiological parameters included distal motor-nerve-conduction of the ulnar nerve measured as distal-motor-latency (dml) from stimulation at proximal wrist to the principal target muscles of the deep motor branch: 1) first dorsal-interosseus-muscle (IOD I), as most distal target muscle, and 2) abductor digiti minimi muscle. Weakness of ulnar innervated hand muscles was evaluated by using the Medical Research Council (MRC) scale 0–5. In GCS hypoesthesia/paresthesia is typically missing or a less dominant finding confined to the distal ulnar aspect of the hand. Sensory function was examined by evaluating and scoring two-point discrimination, pain and proprioception (0 = no sensory deficits, 1 = mild hypoesthesia/paresthesia, 2 = severe hypoesthesia/paresthesia). According to distal ulnar branching variants, clinical symptoms may appear differently in GCS: varying degrees of both, motor and sensory deficits in case of ulnar nerve trunk injury (Zone I); pure motor deficits (the predominant clinical presentation) in case of isolated injury to the deep motor branch (Zone II); and pure sensory disturbances in case of isolated affection of the superficial sensory branch (Zone III).

### IV. MRI Protocol

MRI examinations were all performed at 3 Tesla magnetic field strength (Magnetom VERIO, Siemens AG, Erlangen/Germany) with the following protocol:

- High-resolution T2-weighted fat-suppressed turbo-spin-echo sequence:
- TR/TE 3830/60 ms, slice thickness 2.0 mm, number of slices 32, voxel-size 0.2×0.2×2.0 mm, intersection gap 0.0 mm, time-of-acquisition 7∶10 min.
- T1-weighted turbo-spin-echo sequences:
- TR/TE 916/20 ms, slice thickness 2.0 mm, number of slices 32, voxel-size 0.2×0.2×2.0 mm, intersection gap 0.0 mm, time-of-acquisition 6∶12 min.

Patients were examined in prone position with the arm extended in pronation and the hand placed in an 8-channel surface array coil (Invivo, Siemens AG, Erlangen, Germany). The palpable eminence of the pisiform bone was placed in the center of the wrist coil. To avoid an artificial increase in nerve T2-signal by the Magic Angle effect [37], the arm was aligned at an angle of <10° relative to the B_0_ field direction. Although a markedly increased nerve T2-signal within the ulnar nerve trunk can be detected at a field strength of 1.5 Tesla too, a 3.0 Tesla scanner was necessary to achieve high structural and fascicular resolution even in the small distal nerve branches.

### V. Image and Statistical Analysis

Image analyses were performed on a Siemens-Syngo-Workstation (version VE31A) by two independent experienced radiologists blinded to clinical data (J.K. and M.P). Patients and volunteers were anonymized and assigned to analyses in random order by other investigators (PB, DM). Nerve T2-signal at the wrist was evaluated within the ulnar nerve trunk, the deep, and the superficial branch, as well as within the median nerve by manually delineating nerve circumference as intraneural region of interest (iROI) on each axial imaging slice. To make sure that nerve T2-signal was not artificially influenced by signal from small vessels, accurate transaxial visual inspection was necessary for all high-resolution T2-w image sections. In this manner, small epineurial and intraneural vessels could be reliably identified by their tubular, winded course and their homogeneous strong T2-signal increase. True lesions of nerve fascicles, however, strictly follow a straight course along the peripheral nerve usually with a less intense T2-signal increase. To determine contrast-to-noise ratios, additional ROIs were placed in the thenar muscles (mROI) as well as in the air (standard deviation of air, SD_air_).
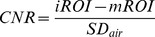
Furthermore, we analyzed the nerve caliber by pixel-wise measurement of cross-sectional nerve area on the T2-weighted images.

Balanced pairwise comparisons were accomplished with Student’s t-test, unbalanced comparisons with Wilcoxon’s signed-rank test (Stata SE 11; Stata, College Station, Tex). Receiver-operating-characteristic (ROC) analyses were performed with R-language of statistical computing (version 2.11.1). All statistical analyses were performed by J.K. and M.P.

## Results

### ROC Analysis of T2-signal in Ulnar Nerve Trunk, Motor and Sensory Branch, and Median Nerve

**Figure 1:**
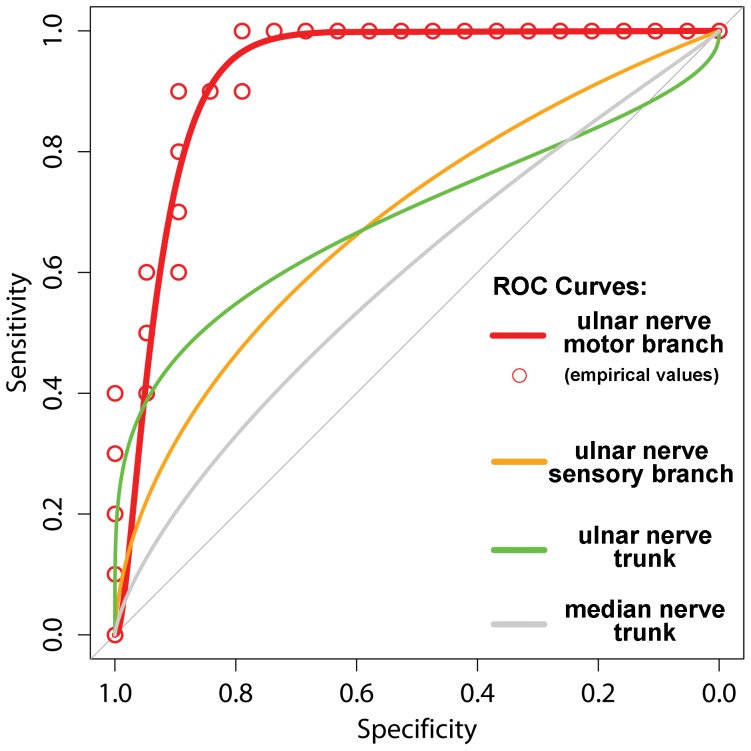
ROC plots of sensitivity versus specificity for the discrimination between GCS and healthy status. Nerve T2-signal was evaluated for its performance of diagnostic classification, empirical and fitted values are plotted. High diagnostic performance (AUC = 0.94) for nerve T2-signal of the deep motor branch was observed, while diagnostic of the ulnar nerve trunk (AUC = 0.70) and the superficial branch were significantly inferior (AUC 0.69).

ROC curves and ROC measures of diagnostic performance were obtained for ulnar nerve trunk, motor and sensory branch of the ulnar nerve and for the median nerve serving as asymptomatic control nerve (Figure 1). The results show that an increase of nerve T2-signal of the deep motor branch determines GCS with strongest diagnostic performance: (AUC 0.94, 95% CI: 0.85–1.00). Sensitivity and specificity values depend on the T2-CNR cut-off value which at a T2-CNR cut-off value of 2.4 revealed a sensitivity of 90.0% and specificity of 89.5%, respectively. ROC/diagnostic performance was significantly inferior for ulnar nerve trunk (AUC 0.70, 95% CI: 0.45–0.95) on pairwise AUC comparison (ulnar nerve trunk vs. deep motor branch, p<0.05). It was also significantly inferior for the sensory ulnar branch (AUC 0.69, 95% CI: 0.46–0.93) on pairwise AUC comparison (sensory branch vs. deep motor branch, p<0.05). It was lowest for the median nerve (AUC 0.58, 95% CI: 0.35–0.82) and significantly inferior on pairwise AUC comparison (median nerve vs. deep motor branch of ulnar nerve, p<0.05).

### Anatomical Proximal-to-distal Mapping of Nerve T2-signal in Patients and Controls: Ulnar Nerve Trunk and Median Nerve Trunk (Internal Control)

The ulnar nerve trunk was identified best and evaluated from proximal to distal on slice positions -9 mm (proximal) to 0 mm (distal) referenced to the center of the pisiform bone.

**Figure 2:**
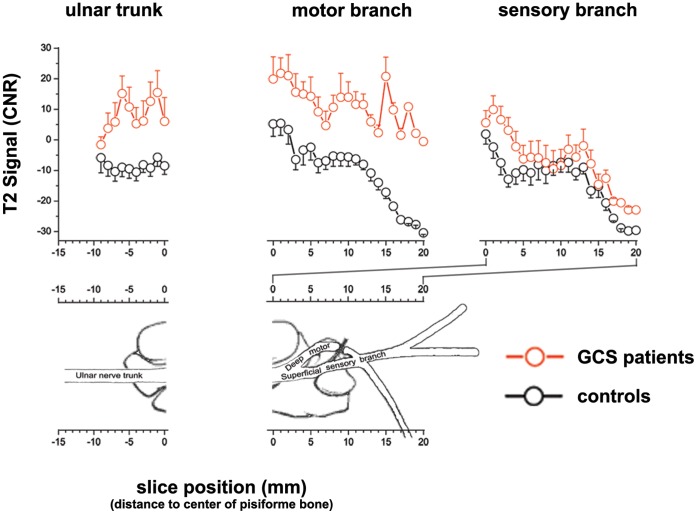
Proximal-to-distal mapping of nerve T2-signal. Mean T2-weighted CNR values and corresponding SEM (error bars) for the ulnar nerve trunk (left), the deep motor (middle) and the superficial sensory (right) branches. Graphs are plotted separately for GCS patients (red lines and dots) and asymptomatic controls (black lines and dots). Note the strong, statistically significant difference (p = 0.001) of increased T2-signal within the deep motor branch just distal to the osseus center of the pisiform bone for GCS patients as compared to healthy controls. T2-signal of the ulnar trunk more than the sensory branch was increased likewise compared to controls but without statistical significance. Median nerve T2-signal within each subject was not any different from ulnar nerve T2-signal (given in text of results section, not shown here). On the lower half of this graph array, an anatomical schematic drawing (according to Shea and McClain, 1969, with permission from The Journal of Bone and Joint Surgery, Volume 51, pages 1095–1103) is supposed to indicate the anatomical position of each slice with reference to the osseous center of the pisiform bone (slice position  = 0) as anatomical landmark. Slices proximal to center were assigned negative numbers indicating the distance (in mm) to center, slices distal to center were assigned positive numbers.

Overall, mean T2-signal (T2-weighted CNR values) of the ulnar trunk in Guyon’s canal (slice positions -9 to 0) was higher in patients with GCS than in asymptomatic volunteers, but this difference remained without statistical significance (GSC: mean T2-CNR 6.8±6.4 SE, controls: mean CNR −7.4±2.5 SE; p = 0.07; figure 2). As an internal/intrasubject control, nerve T2-signal of the median nerve was additionally analyzed from proximal-to-distal showing no difference between GCS patients and healthy volunteers (GCS: mean CNR −6.7±4.2 SE; controls: mean CNR −9.8±2.5 SE; p = 0.45).

### Anatomical Proximal-to-distal Mapping of Nerve T2-signal: Deep Motor and Superficial Sensory Branches of Distal Ulnar Nerve

The deep motor branch as well as the superficial sensory branch of the ulnar nerve was evaluated from slice positions +1 mm (proximal) to +20 mm (distal).

**Figure 3:**
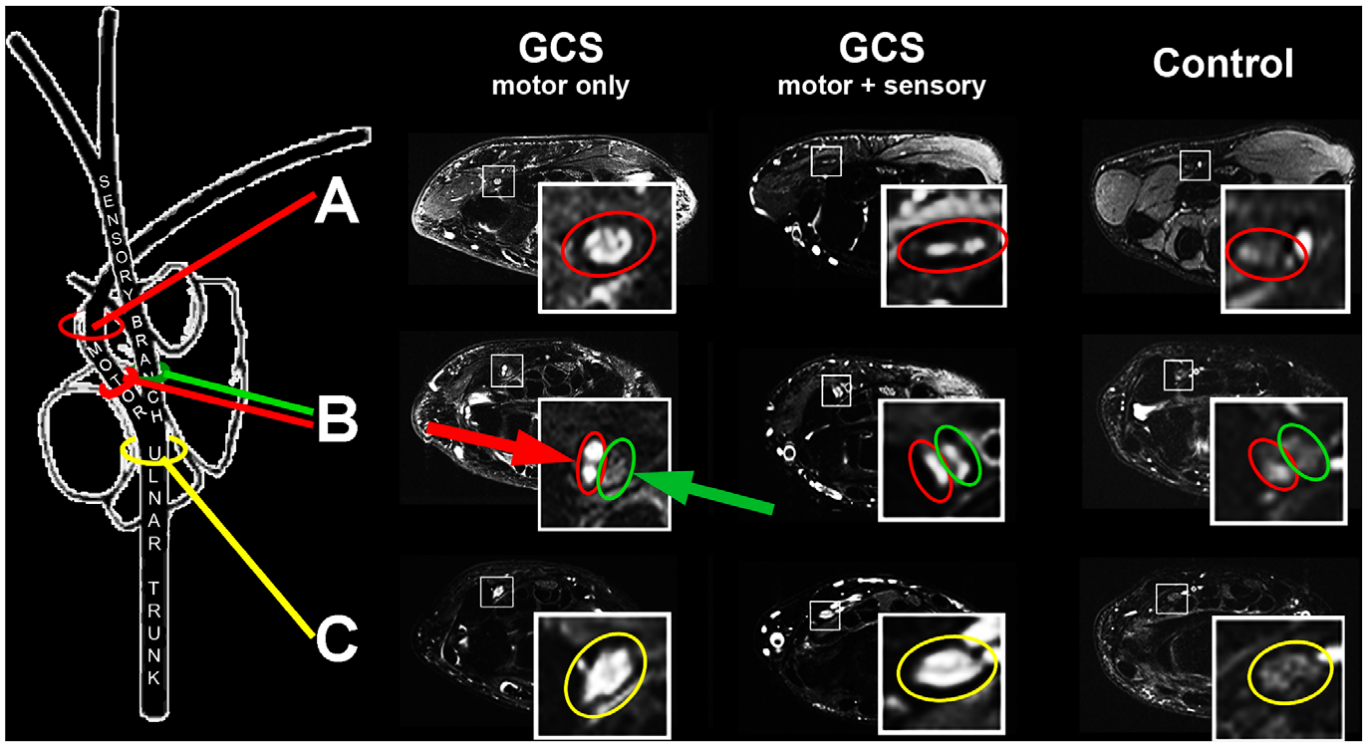
Representative findings of nerve T2-signal increase on single subject level. one GCS patient with isolated motor symptoms (left column), another with combined motor and sensory symptoms (middle column), both compared to one asymptomatic control (right column). Three image sections from distal (A) to proximal (C) are given and image positions indicated on the anatomical schematic drawing on the left (with permission from The Journal of Bone and Joint Surgery, Volume 51, pages 1095–1103). The deep motor branch at the level of the hook of the hamate is encircled in red and found in the upper image row (A = slice position 10). Just distal to the bifurcation of the ulnar nerve motor and sensory branches are encircled in red and green, respectively (middle image row, B, slice position +3). Just proximal to the bifurction the ulnar nerve trunk is encircled in yellow (lower image row, B = slice position -3). In GCS with isolated motor symptoms (left column, GCS motor only) increased nerve T2-signal was most noticeable within the deep motor branch (red arrow) and extended over a short distance proximally into the ulnar nerve trunk (yellow circle in C), while the T2-signal was normal within the superficial sensory branch (green arrow). In case of combined motor and sensory symptoms both distal ulnar nerve branches clearly show an incresed nerve branch T2-signal (red and green circle without arrows in level B, middle column, GCS motor+sensory).

Mean T2-signal of the deep motor branch was strongly increased from proximal-to-distal. This difference was statistically significant (GCS: mean CNR 11.7±4.8 SE; controls: mean CNR -5.3±2.4 SE; p = 0.001; figure 2). Mean T2-signal of the superficial sensory branch likewise exhibited slightly higher values in patients than in controls but without statistical significance (GCS: mean CNR -2.1±4.9 SE; controls: mean CNR -9.7±2.9 SE; (p = 0.08; figure 2). Furthermore, as demonstrated on representative MR images in figure 3, we could show that the anatomical distribution of T2-signal increase corresponds closely to clinical symptoms. In isolated motor GCS, T2-signal within the deep motor branch is strongly increased, whereas proximal T2-signal increase within the ulnar nerve trunk occurs with short extension not reaching the sensory fascicles. However, in combined motor and sensory GCS, T2-signal increase strongly extends distally involving sensory fascicles, too.

GCS is not reflected by caliber increase of ulnar nerve trunk or its distal branches.

Only small, statistically non-significant differences in nerve caliber were found between both groups for the ulnar nerve trunk (asymptomatic controls: 160.7±8.9 vs. GCS patients: 174.4±22.4; p = 0.54), for the deep motor branch (72.3±5.4 vs. 80.4±25.4; p = 0.54) and for the superficial sensory branch (88.4±8.1 vs. 80.7±13.3; p = 0.48).

### Clinical and Electrophysiological Findings

All patients exhibited motor deficits on clinical examination with predominant affection of the IOD I muscle (median MRC IOD I score 2). 6 patients presented with additional typical sensory deficits as described above (3 patients suffered from mild hypoesthesia or paresthesia (scored 1), 3 experienced severe symptoms (scored 2)). Electrophysiological results revealed an increase in motor-nerve-conduction time which was most pronounced for the most distal target muscle of the ulnar nerve, the IOD I (measured as distal-motor-latency; mean 5.85±0.66 SE, range from 3.0–10.3 ms; Table 1 1–11). Within the group of asymptomatic controls, 6 participants (12 wrists) in addition to the MRI study examination also volunteered for electrophysiological examination (mean dml IOD I 3.28±0.10, range 2.7–3.8; mean dml abductor digiti minimi muscle 2.86±0.11, range 2.2–3.3). Clinical as well as electrophysiological examination of healthy volunteers was without pathological findings (Table 1. 12–23).

**Table 1:** Patient (No. 1–11) and volunteer data (No. 12–23) with detailed clinical and electrophysiological findings.

No.	Age/Sex	Hypesthesia (0–2)	Strenght (MRC 0–5)		Elektrophysiology	
			Dorsal interosseus I	Hypothenar	DML^1^	DML^2^
**GCS patients**						
1	26/F	2	1	1	5.3 ms	2.7 ms
2	53/F	1	4	4	5.2 ms	3.3 ms
3	55/M	2	2	2	6.5 ms	3.0 ms
4	43/M	0	2	5	4.5 ms	3.3 ms
5	63/M	1	2	3	4.5 ms	-
6	24/F	0	3	5	5.0 ms	2.9 ms
7	68/M	1	2	3	7.9 ms	3.7 ms
8	49/F	0	4	5	3.0 ms	2.8 ms
9	46/F	2	3	3	10.3 ms	3.6 ms
10	59/F	0	5	3	7.3 ms	3.3 ms
11	74/F	0	2	5	4.8 ms	2.5 ms
**Healthy controls**						
12	34/M	0	5	5	3.8 ms	3.2 ms
13	34/M	0	5	5	3.5 ms	2.8 ms
14	26/F	0	5	5	3.1 ms	2.5 ms
15	26/F	0	5	5	2.8 ms	2.7 ms
16	29/F	0	5	5	3.5 ms	3.0 ms
17	29/F	0	5	5	3.7 ms	3.2 ms
18	33/M	0	5	5	3.3 ms	3.2 ms
19	33/M	0	5	5	3.2 ms	3.0 ms
20	25/F	0	5	5	3.1 ms	2.4 ms
21	25/F	0	5	5	3.3 ms	2.2 ms
22	33/M	0	5	5	2.7 ms	2.8 ms
23	33/M	0	5	5	3.3 ms	3.3 ms

Hypothenar includes the following muscles: abductor digiti minimi muscle, flexor digiti minimi muscle and opponens digiti minimi muscle.

DML^ 1^: distal-motor-latency to IOD I.

DML^ 2^: distal-motor-latency to abductor digiti minimi muscle.

**Figure 4:**
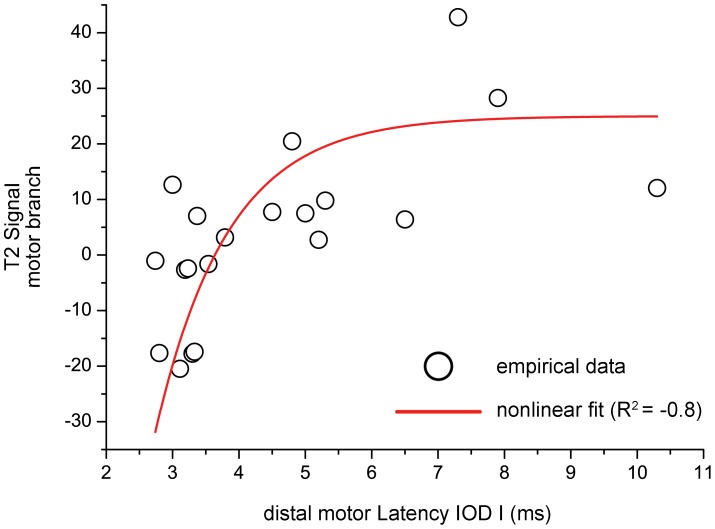
Correlation between deep motor branch T2-signal (y-axis) and its electrical conduction time in ms (x-axis). Electrical conduction time through the deep motor branch was measured as distal motor latency to its most distal target muscle which is the first dorsal interosseus muscle (IOD I). Note how the empirical data (black dots) strongly correlate (R^2^ = −0.8; p<0.001) in a non-linear fashion (non-linear fit of asymptotic growth, red dashed line). This finding indicates that nerve T2-signal and electrical nerve conduction show a distinctive behavior under physiological and pathological condition, in that the former exhibits its largest dynamic range near the normative reference value of dml IOD I (which is given in the literature at 3–5 ms), and that electrical conduction exhibits its largest dynamic range in severe pathological states (>5 ms). This may point toward a strong diagnostic value of nerve T2-signal as a novel marker for the early detection of nerve injury.

Electrical nerve conduction of the deep motor branch measured as the time from stimulation at the proximal wrist to excitation of motor potentials of its most distal target muscle (dml to IOD I) showed a significant non-linear correlation with motor branch T2-signal (R^2^ = −0.8; p<0.001). Motor branch T2-signal increased steeply within the range of beginning deceleration of electrical conduction (3–5 m/s) until reaching a plateau within the range of severe electrical conduction slowing (>5 m/s). Figure 4 shows empirical and fitted data of deep motor branch T2-signal plotted against electrical conduction through this same distal ulnar nerve branch measured as dml to the IOD I muscle.

## Discussion

Nerve T2-signal has been suggested as a potential *direct* diagnostic sign to determine a peripheral nerve lesion in focal and non-focal neuropathies [9]–[10], [16], [17], [19], [22]. *Direct* imaging signs would be helpful to indicate and localize nerve lesions, even in the absence of *indirect* imaging findings such as muscle denervation or mass lesions inside or adjacent to peripheral nerves [4], [15], [38].

To explore the diagnostic value of nerve T2-signal in GCS, a defined patient population (so-called index population) confirmed with GCS by clinical and electrophysiological criteria was evaluated and compared to asymptomatic controls [39]. With this design it is not intended to compare diagnostic performance of T2-signal with established clinical and electrophysiological diagnostic criteria, but to test if nerve T2-signal is a potentially useful diagnostic marker reflecting clinical symptoms and/or electrophysiological alterations to a degree that is measurable and stable at all.

We report here on the first diagnostic imaging study investigating nerve T2-signal in distal ulnar nerve branches at the wrist which are at the limit of spatial resolution even with current coil and MRI technology at high magnetic field strength of 3 Tesla. In patients with confirmed GCS (index population) we found a strong and statistically significant T2-signal increase of the deep motor branch in comparison to asymptomatic controls. The difference of T2-signal between patients and controls was strong and reliable which is reflected by the results of ROC analysis yielding significant diagnostic discrimination between both groups: AUC of 0.94, sensitivity 90%, specificity 89.5%. A diagnostic test performing in the range of 90% sensitivity/specificity with an AUC value of 0.94 usually is attributed to be an *“excellent*” diagnostic test. Still it has to be emphasized that GCS was already determined by clinical and electrophysiological criteria in our study, so that sensitivity/specificity might actually be lower in patients with a merely suspected entrapment neuropathy. Furthermore, our study was performed as a single center study, and therefore only a restricted small sample size could be recruited. Although GCS is the second most frequent entrapment neuropathy of the ulnar nerve, it is per se a rare entrapment neuropathy. All patients presenting with GCS between January 2010 and May 2011 could be recruited. However, our results support that nerve T2-signal increase, even within the fine ulnar nerve branches at the wrist, seems to be strongly associated with clinically manifest neuropathy. Anatomical proximal-to-distal mapping of nerve branch T2-signal showed that T2-signal increase was strongest for the deep motor branch, which is consistent with a predominant presentation of motor deficits in GCS. Nevertheless, T2-signal in the ulnar trunk just proximal to the bifurcation into its distal branches, and also in the sensory branch was elevated in comparison with controls but did not reach statistical significance. This observation can be explained by additional sensory symptoms in 6 of 11 GCS patients in our study. As shown on figure 3, on single subject level qualitative visual assessment of T2-signal within the motor and sensory branch could reliably predict if pure motor symptoms or combined motor-sensory deficits were present.

That nerve T2-signal increase reflects underlying clinically relevant pathology is also strongly supported by the results of comparing nerve T2-signal with motor-nerve-conduction through the deep motor branch of the ulnar nerve. To the best of our knowledge, correlation analysis between nerve T2-signal and electrical nerve-conduction as established gold-standard has not been performed before by any human or animal research study. Our results showed that conduction slowing is strongly correlated with T2-signal increase of the deep motor branch. This correlation was found to be non-linear following a function of saturated/asymptotic growth (R^2^ = −0.8; p<0.001, Figure 4), which is particularly striking for two reasons. Firstly, it indicates that nerve T2-signal, still an imaging indicator of unclear pathophysiological significance, is strongly associated with electrical nerve function. Secondly, since T2-signal increases steeply within the lower value range of electrical nerve-conduction slowing, and saturates with more severe conduction slowing, it seems that T2-signal alteration behaves differently than motor conduction slowing, at least in entrapment injury as studied here. According to these results, it may be cautiously interpreted that T2-signal seems to have a particularly strong sensitivity to detect mild nerve injury, because under such mild pathological condition it increases more dynamically than electrical motor-nerve-conduction slows down. Conduction slowing, on the other hand, is more dynamic under the condition of more severe injury (Figure 4). This particular behavior of nerve T2-signal in entrapment nerve injury, at vulnerable anatomical sites, may carry the risk of producing false positive results. This is supported by finding the peak of T2-signal in the center of Guyon’s canal in patients as well as in asymptomatic controls, but in the latter to a significantly lower degree and with less proximal-to-distal extension (Figure 2). A slightly increased ulnar nerve T2-signal in asymptomatic subjects is not restricted to GCS but was found in cubital tunnel at the elbow, too [9], [40]–[42]. An underlying artificial increase of nerve T2-signal by the so-called magic angle effect, was excluded in this study by aligning the extended forearm at an angle of <10° relative to the B_0_ field direction during all MR examinations [37], [43]. Therefore, the correct determination of a true nerve lesion has to be made cautiously and the proximal-to-distal extension and intensity of nerve T2-signal has to be taken into account. Furthermore, follow up MRI measurements after either conservative or surgical treatment were not performed but would be necessary to clarify if T2-signal increase was partly or completely reversible and paralleled clinical recovery.

In summary, our results demonstrate that monitoring nerve T2-signal of distal ulnar nerve branches within Guyon’s canal allows precise determination of GCS and strongly correlates with electrical nerve-conduction, the current diagnostic gold-standard. These findings suggest that nerve T2-signal may be a useful novel diagnostic marker which may become relevant especially in cases in which the exact site of nerve entrapment cannot be clearly determined otherwise.
